# Genome sequence of the *Wenxinia marina* type strain (DSM 24838^T^), a representative of the *Roseobacter* group isolated from oilfield sediments

**DOI:** 10.4056/sigs.5601028

**Published:** 2014-03-28

**Authors:** Thomas Riedel, Anne Fiebig, James Han, Marcel Huntemann, Stefan Spring, Jörn Petersen, Natalia N. Ivanova, Victor Markowitz, Tanja Woyke, Markus Göker, Nikos C. Kyrpides, Hans-Peter Klenk

**Affiliations:** 1 Sorbonne Universités, UPMC Univ Paris 06, USR 3579, LBBM, Observatoire Océanologique, Banyuls/Mer, France; 2 CNRS, USR 3579, LBBM, Observatoire Océanologique, Banyuls-sur-Mer, France; 3 Leibniz Institute DSMZ – German Collection of Microorganisms and Cell Cultures, Braunschweig, Germany; 4 DOE Joint Genome Institute, Walnut Creek, California, USA; 5 Biological Data Management and Technology Center, Lawrence Berkeley National Laboratory, Berkeley, California, USA

**Keywords:** aerobic, heterotrophic, rod-shaped, quorum sensing, autoinducer, prophage-like structures, *Roseobacter* group, *Rhodobacteraceae*, *Alphaproteobacteria*

## Abstract

*Wenxinia marina* Ying *et al.* 2007 is the type species of the genus *Wenxinia*, a representative of the *Roseobacter* group within the alphaproteobacterial family *Rhodobacteraceae*, isolated from oilfield sediments of the South China Sea. This family was shown to harbor the most abundant bacteria especially from coastal and polar waters, but was also found in microbial mats, sediments and attached to different kind of surfaces.

Here we describe the features of *W. marina* strain HY34^T^ together with the genome sequence and annotation of strain DSM 24838^T^ and novel aspects of its phenotype. The 4,181,754 bp containing genome sequence encodes 4,047 protein-coding genes and 59 RNA genes. The genome of *W. marina* DSM 24838^T^ was sequenced as part of the activities of the Genomic Encyclopedia of Type Strains, Phase I: the one thousand microbial genomes (KMG) project funded by the DoE and the Transregional Collaborative Research Centre 51 (TRR51) funded by the German Research Foundation (DFG).

## Introduction

Strain HY34^T^ (= DSM 24838^T^ = CGMCC 1.6105^T^ = JCM 14017^T^) is the type strain of *Wenxinia marina* in the monospecific genus *Wenxinia* [1,2], which belongs to the widely distributed marine *Roseobacter* group [3]. The strain was isolated from sediments of the Xijiang oilfield located in the South China Sea (China) [1]. The genus *Wenxinia* was named after Professor Wen-Xin Chen, a Chinese pioneer in soil microbiology. The species epithet *marina* refers to the Latin adjective *marina* (‘of or belonging to the sea’) [1,2]. Current PubMed records do not indicate any follow-up research with strain HY34^T^ after the initial description of *W. marina* [1].

In this study we analyzed the genome sequence of *W. marina* DSM 24838^T^. We present a description of the genome sequencing and annotation and present a summary classification together with a set of features for strain HY34^T^, including novel aspects of its phenotype.

## Classifications and features

### 16S rRNA gene analysis

**Figure 1:**
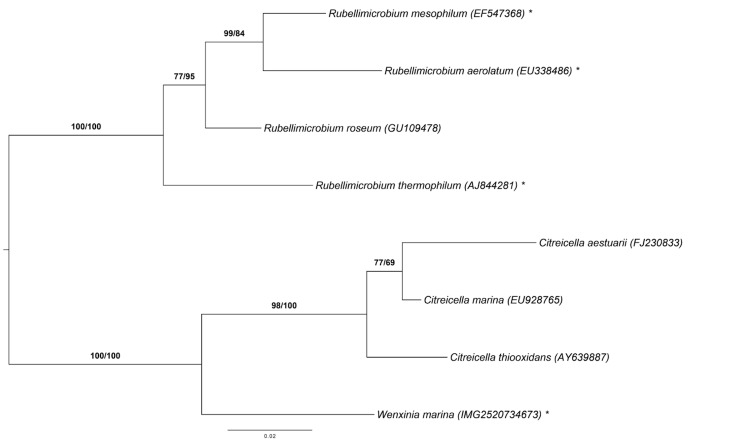
Phylogenetic tree highlighting the position of *W. marina* relative to the type strains of the neighboring genera *Citreicella* and *Rubellimicrobium*. The tree was inferred from 1,381 aligned characters of the 16S rRNA gene sequence under the maximum likelihood (ML) criterion as previously described [4]. The branches are scaled in terms of the expected number of substitutions per site. Numbers adjacent to the branches are support values from 1,000 ML bootstrap replicates (left) and from 1,000 maximum-parsimony bootstrap replicates (right) if larger than 60% [4]. Lineages with type strain genome sequencing projects registered in GOLD [5] are labeled with one asterisk, those also listed as 'Complete and Published' with two asterisks [6].

Figure 1 shows the phylogenetic neighborhood of *W. marina* in a 16S rRNA gene based tree. The sequences of the two identical 16S rRNA gene copies in the genome, differ by three nucleotides from the previously published 16S rRNA gene sequence (DQ640643).

A representative genomic 16S rRNA gene sequence of *W. marina* DSM 24838^T^ was compared with the Greengenes database for determining the weighted relative frequencies of taxa and (truncated) keywords as previously described [4]. The most frequently occurring genera were *Ruegeria* (41.6%), *Paracoccus* (31.0%), *Oceanicola* (14.0%), *Silicibacter* (5.0%) and *Loktanella* (3.3%) (60 hits in total). Among all other species, the one yielding the highest score was *Oceanicola granulosus* (AAOT01000021), which corresponded to an identity of 94.7% and an HSP coverage of 99.6%. (Note that the Greengenes database uses the INSDC (= EMBL/NCBI/DDBJ) annotation, which is not an authoritative source for nomenclature or classification.) The highest-scoring environmental sequence was DQ640643 (Greengenes short name '*Rhodobacteraceae* South China Sea oil field sediment isolate HY34 *Rhodobacteraceae* str. HY34'), which showed an identity of 99.8% and an HSP coverage of 100.0%. The most frequently occurring keywords within the labels of all environmental samples that yielded hits were 'microbi' (4.3%), 'coral' (3.6%), 'sea' (2.6%), 'diseas' (2.5%) and 'china' (2.4%) (190 hits in total). The most frequently occurring keywords within the labels of those environmental samples which yielded hits of a higher score than the highest scoring species were 'antecubit, fossa, skin' (13.9%) and 'china, field, oil, rhodobacteracea, sea, sediment, south' (8.3%) (3 hits in total). Some of these keywords fit well to the isolation site of strain HY34^T^ [1].

### Morphology and physiology

**Figure 2:**
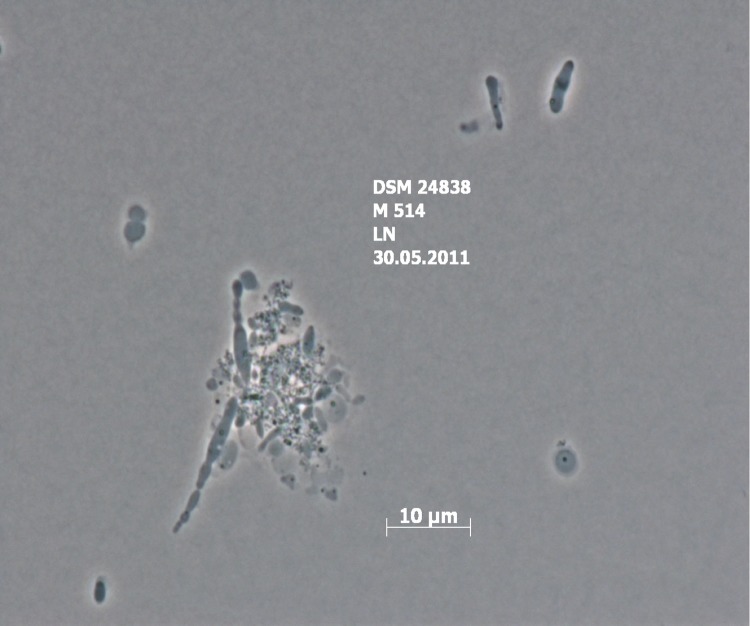
Phase contrast micrograph of *W. marina* DSM 24838^T^.

Cells of strain HY34^T^ form Gram-negative, ovoid or short rods (0.7-0.8 µm in width and 1.3 µm in length) [Figure 2]. Motility and sporulation were not observed. Cells are strictly aerobic and display a heterotrophic lifestyle. When cultured on Marine Agar 2216 colonies with a weak pink color became visible, but bacteriochlorophyll *a* was not detected. The strain grows in a temperature range of 15-42°C with an optimum at 34-38°C. NaCl is required for growth (0.5-9%) with an optimum salt concentration at 1-4%. Further, the strain grows in a range of pH 6.5-8.5 with an optimum pH of 7.5-8.0. The strain is oxidase- and catalase-positive. Nitrate is reduced to nitrite. Indole and H_2_S are not produced. Cells hydrolyze urea and Tween 20, and a weak hydrolysis of Tween 40 and Tween 80 was also detected. The strain does not hydrolyze agar, casein, starch, DNA or CM-cellulose. Strain HY34^T^ accumulates polyhydroxyalkanoates in its cells. Tests for arginine dehydrolase and lecithinase were negative. Further, cells utilize sucrose, lactose, galactose, maltose, melezitose, L-rhamnose, L-fucose, trehalose, cellobiose, gluconate, lactic acid, malate, L-glutamic acid. The strain utilizes D-melibiose, inulin, methyl α-D-glucoside, glycerol, sorbitol, butanol, pyruvate, formic acid, L-alanine and L-proline weakly. Utilization of D-raffinose, mannitol, L-sorbose, dulcitol, adonitol, *myo*-inositol, methanol, ethanol, citrate, malonate, butyric acid and caprate acid was not detected. Cells produce acid from D-xylose, cellobiose, lactose, L-rhamnose, L-arabinose, D-raffinose, and weakly from sucrose, maltose, mannose, trehalose, and ribose. Strong activities for esterase (C8) and *α-* and *β*-glucosidases were detected, as well as weak activities for alkaline phosphatase, leucine arylamidase, valine arylamidase and naphthol-AS-BI-phosphohydrolase. No activity was found for acid phosphatase, *N*-acetyl-*β*-cysteine arylamidase, glucosamidase, *α*- and *β*-galactosidase, *α*-mannosidase, *α*-chrymotrypsin, *β*-glucuronidase, *α*-fucosidase and lipase (C_14_). Cells of strain HY34^T^ are found to be resistant to norfloxacin, tetracycline and gentamicin as well as sensitive to neomycin, polymyxin B, streptomycin, ampicillin, carbenicillin, vancomycin, ciprofloxacin, rifampicin, chloramphenicol, benzylpenicillin, kanamycin, and erythromycin (all data from [1] and presented in Table 1).

**Table 1:** Classification and general features of *W. marina* HY34^T^ according to the MIGS recommendations [41] (published by the Genomic Standards Consortium [42]).

**MIGS ID**	**Property**	**Term**	**Evidence code**
		Domain *Bacteria*	TAS [43]
		Phylum *Proteobacteria*	TAS [44]
		Class *Alphaproteobacteria*	TAS [45,46]
	Current classification	Order *Rhodobacterales*	TAS [46,47]
		Family *Rhodobacteraceae*	TAS [48]
		Genus *Wenxinia*	TAS [1]
		Species *Wenxinia marina*	TAS [1]
		Strain HY34^T^	TAS [1]
	Gram stain	negative	TAS [1]
	Cell shape	ovoid or short rods	TAS [1]
	Motility	non-motile	TAS [1]
	Sporulation	non-spore-forming	TAS [1]
	Temperature range	15-42°C	TAS [1]
	Optimum temperature	34-38°C	TAS [1]
	Salinity	0.5-9% (NaCl)	TAS [1]
MIGS-22	Oxygen requirement	aerobic	TAS [1]
	Carbon source	Yeast extract, peptone	TAS [1]
	Energy metabolism	heterotroph	TAS [1]
MIGS-6	Habitat	Oilfield sediment	TAS [1]
MIGS-15	Biotic relationship	Free living	TAS [1]
MIGS-14	Pathogenicity	None	NAS
	Biosafety level	1	TAS [49]
MIGS-23.1	Isolation	Oilfield sediment	TAS [1]
MIGS-4	Geographic location	Xijiang oilfield, South China Sea (China)	TAS [1]
MIGS-5	Sample collection time	before 2007	NAS
MIGS-4.1	Latitude	Not reported	
MIGS-4.2	Longitude	Not reported	
MIGS-4.3	Depth	100 m	TAS [1]
MIGS-4.4	Altitude	Not reported	

Evidence codes - TAS: Traceable Author Statement (i.e., a direct report exists in the literature); NAS: Non-traceable Author Statement (i.e., not directly observed for the living, isolated sample, but based on a generally accepted property for the species, or anecdotal evidence). Evidence codes are from of the Gene Ontology project [50].

The utilization of carbon compounds by *W. marina* DSM 24838^T^ grown at 28°C was also determined for this study using Generation-III microplates in an OmniLog phenotyping device (BIOLOG Inc., Hayward, CA, USA) [7]. The microplates were inoculated with a cell suspension at a cell density of 95-96% turbidity and dye IF-A. Further additives were vitamin, micronutrient and sea-salt solutions, which had to be added for dealing with such marine bacteria [8]. The plates were sealed with parafilm to avoid a loss of fluid.

The exported measurement data were further analyzed with the opm package for R [9,10], using its facilities for statistically estimating parameters from the respiration curves such as the maximum height, and automatically translating these values into negative, ambiguous, and positive reactions. The reactions were recorded in three biological replicates.

On the Generation-III plates, the strain was positive for pH 6, 1% NaCl, 4% NaCl, 8% NaCl, D-galactose, 3-O-methyl-D-glucose, D-fucose, L-fucose, L-rhamnose, 1% sodium lactate, myoinositol, rifamycin SV, L-aspartic acid, L-glutamic acid, L-histidine, L-serine, D-glucuronic acid, quinic acid, L-lactic acid, citric acid, *α*-keto-glutaric acid, D-malic acid, L-malic acid, nalidixic acid, and sodium formate.

*W. marina* HY34^T^ was negative for the following tests*:* dextrin, D-maltose, D-trehalose, D-cellobiose, *β*-gentiobiose, sucrose, D-turanose, stachyose, pH 5, D-raffinose, α-D-lactose, D-melibiose, *β*-methyl-D-galactoside, D-salicin, *N*-acetyl-D-glucosamine, *N*-acetyl-*β*-D-mannosamine, N-acetyl-D-galactosamine, *N*-acetyl-neuraminic acid, D-glucose, D-mannose, D-fructose, inosine, fusidic acid, D-serine, D-sorbitol, D-mannitol, D-arabitol, glycerol, D-glucose-6-phosphate, D-fructose-6-phosphate, D-aspartic acid, D-serine, troleandomycin, minocycline, gelatin, glycyl-L-proline, L-alanine, L-arginine, L-pyroglutamic acid, lincomycin, guanidine hydrochloride, niaproof, pectin, D-galacturonic acid, L-galactonic acid-*γ*-lactone, D-gluconic acid, glucuronamide, mucic acid, D-saccharic acid, vancomycin, tetrazolium violet, tetrazolium blue, phydroxy-phenylacetic acid, methyl pyruvate, D-lactic acid methyl ester, bromo-succinic acid, lithium chloride, potassium tellurite, tween 40, *γ*-amino-n-butyric acid, α-hydroxy-butyric acid, *β*-hydroxy-butyric acid, *α*-keto-butyric acid, acetoacetic acid, propionic acid, acetic acid, aztreonam, butyric acid and sodium bromate.

The phenotype microarray results fit to those reported by Ying and colleagues [1] in large part. Only the utilization of lactose and D-trehalose could not be confirmed by respiration measurements under the given conditions. Interestingly, *W. marina* DSM 24838^T^ showed a varying phenotype both in growth measurement [1] and in the respiration curves among replicates. Ying and colleagues reported eleven substrates yielding “weak” results, which complicates the exact comparison of substrate utilization [1]. In contrast to Ying and colleagues, the OmniLog measurements gave positive reactions for L-histidine and myoinositol. This may be due respiratory measurements being more sensitive than growth measurements [11].

### Chemotaxonomy

The principal cellular fatty acids of strain HY34^T^ are C_18:1 ω7c_ (57.1%), C_16:0_ (16.5%), 11-methyl C_18:1 ω7c_ (5.4%), C_18:0_ (3.9%), C_14:0_ (3.7%), C_15:1 iso G_ and C_15:1 iso I_ (3.4%), summed feature 3 C_16:1 ω7c_ and/or C_15:0 2-OH_ (1.9%), C_12:0_ (1.6%) and C_13:0 2-OH_ (1.2%). The major respiratory lipoquinone was ubiquinone 10, which is a well-known characteristic of the *Alphaproteobacteria*. Phosphatidylglycerol and phosphatidylcholine were identified as the major polar lipids. In contrast to other representatives of the *Roseobacter* group such as *Marinovum algicola* FF3^T^ (DSM 10251^T^) [12,13], strain HY34^T^ also contains an unidentified glycolipid called L1, which shows similarities to an unidentified phospholipid of *Ruegeria atlantica* DSM 5828^T^ (all data from [1]).

## Genome sequencing and annotation

### Genome project history

This strain was twice selected for genome sequencing on the basis of its phylogenetic position [14]. First as part of the DFG funded project “Ecology, Physiology and Molecular Biology of the *Roseobacter* clade: Towards a Systems Biology Understanding of a Globally Important Clade of Marine Bacteria” and later as part of the “Genomic Encyclopedia of Type Strains, Phase I: the one thousand microbial genomes (KMG) project” [15], a follow-up of the GEBA project [16], which aims in increasing the sequencing coverage of key reference microbial genomes. The strain was independently sequenced from the same source of DNA and produced draft sequences that were finally joined. The project information can found in the Genomes OnLine Database [5] and the Integrated Microbial Genomes database (IMG) [17]. A summary of the project information is shown in Table 2.

**Table 2:** Genome sequencing project information

MIGS ID	Property	Term
MIGS-31	Finishing quality	Non-contiguous finished
MIGS-28	Libraries used	Two genomic libraries: one Illumina PE library (539 bp insert size), one 454 PE library (3kb insert size)
MIGS-29	Sequencing platforms	Illumina GA IIx, Illumina MiSeq, 454 GS-FLX Titanium
MIGS-31.2	Sequencing coverage	356 ×
MIGS-30	Assemblers	velvet version 1.1.36, Newbler version 2.3, consed 20.0
MIGS-32	Gene calling method	Prodigal 1.4
	GenBank Date of Release	pending
	GOLD ID	Gi10895
	NCBI project ID	183669
	Database: IMG	2519899719 (8 scaffold version) and 2515154190 (41 scaffold version)
MIGS-13	Source material identifier	DSM 24838^T^
	Project relevance	Tree of Life, biodiversity

### Growth conditions and DNA isolation

A culture of *W. marina* DSM 24838^T^ was grown aerobically in DSMZ medium 514 [18] at 30°C. Genomic DNA was isolated using Jetflex Genomic DNA Purification Kit (GENOMED 600100) following the standard protocol provided by the manufacturer but modified by an incubation time of 60 min, incubation on ice overnight on a shaker, the use of an additional 50 µl proteinase K, and the addition of 100 µl protein precipitation buffer. The DNA is available from the Leibniz-Institute DSMZ through the DNA Bank Network [19].

### Genome sequencing and assembly

The genome sequencing under the DFG funded part of the project was perform as previously described for *Rubellimicrobium thermophilum* [6], with 3.3 million reads delivered by the first run on an Illumina GAII platform. To increase the sequencing depth, a second Ilumina run was performed, providing another 8.1 million reads. The first draft assembly from 9,139,639 filtered reads (median read length 122 nt) resulted in more than 300 contigs. To gain information on the contig arrangement an additional 454 run was performed. The paired-end pyrosequencing jumping library resulted in 158,608 reads, with an average read length of 450 bp. Both draft assemblies (Illumina and 454 sequences) were fractionated into artificial Sanger reads of 1,000 nt in length plus 75 bp overlap on each site. These artificial reads served as an input for the phred/phrap/consed package [20]. In combination the assembly resulted in 265 contigs in 26 scaffolds.

The genome sequencing under the DoE funded part of the project was performed as previously described for *Halomonas zhanjiangensis* [21] also using the Illumina technology [22]. An Illumina Standard shotgun library was constructed and sequenced using the Illumina HiSeq 2000 platform. All general aspects of library construction and sequencing performed at the JGI can be found at [23]. The final assembly for this part of the project resulted in 41 scaffolds covering 4,175,892 bp (ARAY00000000).

The draft sequence from the first (DFG-funded) part was mapped to the permanent draft version ARAY00000000 using minimus2 [24]. By manual editing the number of contigs was reduced to 22 in 8 scaffolds (AONG00000000). The combined sequences provided a 356 × coverage of the genome.

### Genome annotation

Genes were identified using Prodigal [25] as part of the JGI genome annotation pipeline. The predicted CDSs were translated and used to search the National Center for Biotechnology Information (NCBI) nonredundant database, UniProt, TIGR-Fam, Pfam, PRIAM, KEGG, COG, and InterPro databases. Identifications of RNA genes were carried out by using HMMER 3.0rc1 [26] (rRNAs) and tRNAscan-SE 1.23 (tRNAs) [27]. Other non-coding genes were predicted using INFERNAL 1.0.2 [28]. Additional gene prediction analysis and functional annotation was performed within the Integrated Microbial Genomes - Expert Review (IMG-ER) platform [29] CRISPR elements were detected using CRT [30] and PILER-CR [51].

## Genome properties

**Figure 3:**
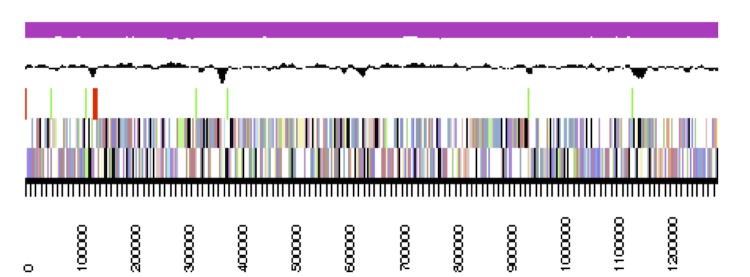
Graphical map of the largest scaffold. From bottom to the top: Genes on forward strand (colored by COG categories), Genes on reverse strand (colored by COG categories), RNA genes (tRNAs green, rRNAs red, other RNAs black), GC content (black), GC skew (purple/olive).

The genome statistics are provided in Table 3 and Figure 3. The genome of DSM 24838^T^ has a total length of 4,181,754 bp and a G+C content of 70.5%. Of the 4,106 genes predicted, 4,047 were protein-coding genes, and 59 RNAs. The majority of the protein-coding genes (80.4%) were assigned a putative function while the remaining ones were annotated as hypothetical proteins. The distribution of genes into COGs functional categories is presented in Table 4.

**Table 3:** Genome Statistics

**Attribute**	Value	% of Total
Genome size (bp)	4,181,754	100.00
DNA coding region (bp)	3,740,397	89.45
DNA G+C content (bp)	2,948,333	70.50
Number of scaffolds	8	
Total genes	4,106	100.00
RNA genes	59	1.44
rRNA operons	2	
tRNA genes	45	1.10
Protein-coding genes	4,047	98.56
Genes with function prediction (proteins)	3,303	80.44
Genes in paralog clusters	3,408	83.00
Genes assigned to COGs	3,199	77.91
Genes assigned Pfam domains	3,379	82.29
Genes with signal peptides	430	10.47
Genes with transmembrane helices	904	22.02
CRISPR repeats	0	

**Table 4:** Number of genes associated with the general COG functional categories

**Code**	**Value**	**%age**	**Description**
J	170	4.9	Translation, ribosomal structure and biogenesis
A	3	0.1	RNA processing and modification
K	203	5.8	Transcription
L	153	4.4	Replication, recombination and repair
B	3	0.1	Chromatin structure and dynamics
D	27	0.8	Cell cycle control, cell division, chromosome partitioning
Y	0	0.0	Nuclear structure
V	40	1.1	Defense mechanisms
T	139	4.0	Signal transduction mechanisms
M	201	5.7	Cell wall/membrane/envelope biogenesis
N	24	0.7	Cell motility
Z	0	0.0	Cytoskeleton
W	0	0.0	Extracellular structures
U	54	1.5	Intracellular trafficking and secretion, and vesicular transport
O	123	3.5	Posttranslational modification, protein turnover, chaperones
C	214	6.1	Energy production and conversion
G	321	9.2	Carbohydrate transport and metabolism
E	372	10.6	Amino acid transport and metabolism
F	78	2.2	Nucleotide transport and metabolism
H	137	3.9	Coenzyme transport and metabolism
I	152	4.3	Lipid transport and metabolism
P	163	4.7	Inorganic ion transport and metabolism
Q	107	3.1	Secondary metabolites biosynthesis, transport and catabolism
R	439	12.5	General function prediction only
S	378	10.8	Function unknown
-	907	22.1	Not in COGs

## Insights into the genome

### Plasmids

Genome sequencing of *W. marina* DSM 24838^T^ reveals the presence of one plasmid with a size of about 101 kb. The plasmid contains a characteristic replicase of the RepA-I type [31], but the typical module structure containing the replicase as well as a *par*AB partitioning operon was not found. A single *par*A gene (wenxma_04096) is located adjacent to the replicase and an additional *par*AB operon (wenxma_04090 to wenxma_04091) is located downstream of *rep*A-I. The plasmid harbors neither a plasmid stability module nor a type-IV secretion system.

The plasmid contains a large RTX-toxin (wenxma_04058) and is dominated by genes that are required for polysaccharide biosynthesis. It includes all four genes of the rhamnose pathway [32], but the *rmlA* gene for the glucose-1-phosphate thymidylyltransferase (EC 2.7.7.24; wenxma_04097) is separated from the remaining clustered genes (*rml*C, *rml*B, *rml*D; wenxma_04094 to wenxma_04092). The extrachromosomal replicon may be involved in surface attachment. Comparable RepA-I type plasmids with a similar genetic composition are also present in other *Rhodobacterales* including several *Phaeobacter* strains [33].

### Phages

Many bacteria encode genome-inserted gene sequences, which are associated with prophages, one of the major reason for horizontal gene transfer and bacterial diversity [34,35]. The genome sequence of *W. marina* DSM 24838^T^ was found to encode several prophage-associated gene sequences (e.g., wenxma_00641 to wenxma_00646, wenxma_00930 to wenxma_00936, wenxma_01496 to wenxma_01510).

### Quorum Sensing

Analysis of the DSM 24838^T^ genome sequence revealed the presence of gene sequences associated to quorum sensing (QS) [36-38]. QS is a bacterial communication system *via* chemical signal molecules called autoinducers, which are produced and released by QS bacteria to coordinate behaviors with respect to their population density [38]. Interestingly and surprisingly, QS induces also individual morphologies and cell division modes, which was recently shown for *D. shibae* DFL-12, another representative of the *Roseobacter* group [39,40]. Regarding to QS the genome of DSM 24838^T^ codes for, e.g., two N-acyl-L-homoserine-lactone synthetases (*Lux*I homologues, wenxma_01086 and wenxma_03269) and two genes possibly encoding QS-involved response and transcriptional regulators (*LuxR* homologues, wenxma_01085 and wenxma_03267).

### Morphological traits

With regard to morphological traits, several genes associated with the putative production, biosynthesis and export of exopopolysaccharides (wenxma_00281, wenxma_02363 and wenxma_02364, wenxma_03720 and wenxma_03721) and capsule polysaccharides (wenxma_00822, wenxma_02023 to wenxma_02025, wenxma_02704 and wenxma_02705, wenxma_04069) were detected.

Interestingly, the genome of DSM 24838^T^ was found to encode several gene sequences putatively involved in pili formation (e.g., wenxma_01776 to wenxma_01787, wenxma_03426 to wenxma_03435) and chemotaxis (e.g., wenxma_3823 to wenxma_03830), although the strain was described as non-motile [1]. Hence, it could be that the formed pili play a role for adhesion or switching-type motility on solid surfaces.

Further, according to its genome strain DSM 24838^T^ accumulates polyhydroxyalkanoates as storage compounds (wenxma_02601 to wenxma_02604), which is in accordance with the findings of Ying and colleagues for strain HY34^T^ [1].
